# Thymidylate Synthase Overexpression Drives the Invasive Phenotype in Colon Cancer Cells

**DOI:** 10.3390/biomedicines10061267

**Published:** 2022-05-29

**Authors:** Wojciech M. Ciszewski, Małgorzata Chmielewska-Kassassir, Lucyna A. Wozniak, Katarzyna Sobierajska

**Affiliations:** 1 Department of Molecular Cell Mechanisms, Medical University of Lodz, Mazowiecka 6/8, 92-215 Lodz, Poland; katarzyna.sobierajska@umed.lodz.pl; 2 Department of Structural Biology, Medical University of Lodz, Zeligowskiego 7/9, 90-752 Lodz, Poland; malgorzata.chmielewska-kassassir@umed.lodz.pl (M.C.-K.); lucyna.wozniak@umed.lodz.pl (L.A.W.)

**Keywords:** TYMS, colon cancer, EMT, metastasis, invasion, MMP-7

## Abstract

Thymidylate synthase (TYMS) is the crucial enzymatic precursor for DNA biosynthesis and, therefore, the critical target for numerous types of chemotherapy, including the most frequently applied agent in colon cancer treatment 5-fluorouracil (5-FU). TYMS also seems to be associated with cancer metastasis and acquiring mesenchymal character by tumor cells during epithelial–mesenchymal transition (EMT). Based on that knowledge, we decided to investigate the role of TYMS in the modulation of invasive ability in colon cancer cells, where its effect on cancer metastasis has not been studied in detail before. We employed colon cancer cells isolated from different stages of tumor development, cells undergoing EMT, and TYMS overexpressing cells. The elongation ratio, cell migration, invasion assay, and MMP-7 secretion were applied to analyze the cell behavior. Important epithelial and mesenchymal markers characteristic of EMT were examined at the protein level by Western blot assay. Overall, our study showed a correlation between TYMS level and invasion ability in colon cancer cells and, above all, a crucial role of TYMS in the EMT regulation. We postulate that chemotherapeutics that decrease or inhibit TYMS expression could increase the effectiveness of the therapy in patients with colon cancer, especially in the metastatic stage.

## 1. Introduction

Colorectal cancer (CRC) is the third most common cancer (11.4% of all cancers), and it is the second leading cause of cancer death in the world (9.4% of all cancer deaths) in both sexes combined [1]. Although significant progress has been made in the diagnosis and treatment of colorectal cancer in recent years, it is estimated that in the next 20 years, the number of deaths due to this cancer will increase 1.8 times [1].

Several chemotherapeutic drugs have been used so far, but 5-fluorouracil (5-FU) is the most common in the treatment of colorectal cancer [2]. 5-FU is an uracil analogue whose action mechanism is based on inhibiting the thymidylate synthase (TYMS) and the erroneous incorporation of fluoropyrimidine into RNA and DNA. 5-FU converts to several active metabolites incorporated into RNA, disrupting its normal processing (maturing rRNA, post-transcriptional modification of tRNA and mRNA splicing) and RNA function [3]. Additionally, 5-FU blocks a synthesis of thymidine required for DNA synthesis by inhibiting TYMS.

Thymidylate synthase is a dimeric protein that synthesizes deoxythymidine monophosphate (dTMP) from deoxyuridine monophosphate (dUMP), using 5,10-methylene-tetrahydrofolate (CH2-THF) as the methyl donor [3]. TYMS has two binding sites, one for CH2-THF and another for nucleotide, which can be stably occupied by 5-FU metabolites. Thus, by preventing binding of the regular substrate dUMP, 5-FU inhibits dTMP synthesis, which leads to dTMP depletion and, consequently, disruption of DNA synthesis and repair, and ultimately to fatal DNA damage [4].

Some studies have connected TYMS expression to cancer development. TYMS upregulation has been observed in various tumors [5,6,7]. It should be noted that the elevated level of TYMS expression was also associated with more invasive and metastatic abilities of cancer cells [6,8,9]. In addition, it has been recently proposed that TYMS is directly involved in the epithelial-to-mesenchymal transition (EMT) of cancer cells [10,11,12].

EMT is a trans-differentiation process that enables the formation of new types of cells and tissues during physiological development (reviewed in detail [13]). However, EMT is also involved in pathological conditions, such as cancer development, that implicates tumor progression and metastasis. During EMT, epithelial cells lose their epithelial morphology and gain mesenchymal ones, causing loss of polarity, cell–cell dissociation, and cell elongation, which increase cell movement and enable tumor cells to escape from primary tumors [14]. Although numerous factors such as cytokines and growth factors can induce EMT, TGF-β and TGF-β-related proteins play a critical role in this process [15]. TGF-β activates EMT through regulation transcription factors from the Snail family, resulting in repression of specific target genes like claudin, E-cadherin, and activation of others, such as N-cadherin and vimentin [16].

Here, we report our studies on the role of TYMS in the modulation of invasive ability in colon cancer cells, where its effect on cancer metastasis is studied in detail. We employed colon cancer cells isolated from different stages of tumor development, cells undergoing EMT, and TYMS overexpressing cells. We have demonstrated that TYMS is involved in the modulation of the EMT and, consequently, in the modulation of colorectal cancer metastasis. Moreover, we have also shown that silencing TYMS expression reverses EMT and inhibits the invasion capacity, which indicates that TYMS as a target for clinical use is warranted.

## 2. Materials and Methods

### 2.1. Cell Lines and Culture

HT-29, LS180, SW60, HCT116, and LoVo cells, isolated from patients with different colon cancer invasive stages, were purchased from American Type Culture Collection Rockville, MD, USA). Stable Snail-transfected LS180 cells (LS180 Snail cl2) and empty vector-transfected LS180 cells (LS180 pcDNA) cells were previously established in our laboratory [17]. Cells were cultured as a monolayer and maintained in DMEM with GlutaMAX™ supplemented with 10% (*v*/*v*) fetal bovine serum (FBS), penicillin (100 U/mL), and streptomycin (100 µg/mL) at 37 °C in a humidified atmosphere with 5% CO_2_. LS180 Snail cl2 cells were also supplemented with geneticin (75 µg/mL). All media and supplements were from Life Technologies (Paisley, UK).

### 2.2. Stable Transfection

The expression vector with TYMS (pcDNA3.1-TYMS) and empty vector (pcDNA3.1) used as a control were introduced into LS180 with Xfect^®^ Transfection Reagent (Clontech, Mountain View, CA, USA) according to the manufacturer’s protocol. Exponentially growing cells were seeded into a 6-well plate and transfected with the pcDNA3.1-TYMS or pcDNA3.1 expression vector. Then, cells were cultured under G418 stress conditions (75 μg/mL) for two weeks. Next, G418-resistant colonies were chosen for sub-cloning and expressions of TYMS protein was confirmed by Western blot. Two stable transfected TYMS (LS180 TYMS cl1 and LS180 TYMS cl3) were selected for further analysis. The changes were examined compared to the empty vector-transfected cells (LS180 pcDNA).

### 2.3. Cell Morphology Analysis

Changes in cell morphology were estimated as described previously [18]. Briefly, five representative images (with average cell density) of exponentially growing cells were captured by EVOS FLoid Cell Imaging Station (Thermo Fisher Scientific, Bothell, WA, USA), and at least 50 cells for each experimental condition were analyzed using ImageJ software version 1.47 (Bethesda, MD, USA).

### 2.4. Transwell Migration and Invasion Assay

Both assays were performed using modified Boyden chambers containing polycarbonate membranes (ThinCert^®^, Greiner, Kremsmünster, Austria). The lower chambers were filled with 0.4 mL of DMEM supplemented with 0.02% fibronectin (Sigma-Aldrich, Darmstadt, Germany; transmigration assay) or 20% FBS (invasion assay). Cells were labeled with CellTracker™ Red CMTPX Dye (Life Technologies, Eugen, OR, USA; according to manufacturers’ instruction) before harvesting and counting. Then, cells were suspended in DMEM serum-free medium, loaded onto the upper chamber (transmigration assay) or chamber pre-coated with Matrigel (Corning, NY, USA; invasion assay) and allowed to migrate or invade at 37 °C in 5% CO_2_ for 6 h (transmigration assay) or 8 h (invasion assay). Then, the upper surface of the membrane was wiped with a cotton-tip applicator to remove any non-migrated cells. The migrated cells were visualized with EVOS FLoid Cell Imaging Station (Thermo Fisher Scientific, Bothell, WA, USA) at 20× magnification and counted from five representative fields using ImageJ software version 1.47 (Bethesda, MD, USA).

### 2.5. MMP-7 ELISA Assay

Matrix Metallopeptidase 7 (MMP-7) secretion was quantified in a medium using Human Total MMP-7 Quantikine ELISA Kit (R&D Systems, Minneapolis, MN, USA) according to the manufacturer’s instruction. The absorbance was measured at 450 nm using an Infinite F50 microplate reader (Tecan, Groding, Austria).

### 2.6. Protein Extraction and Western Blotting

Whole-cell extracts for Western blotting analysis were prepared as described previously [19]. Briefly, following treatment, cells were lysed in M-PER lysis buffer (Thermo Scientific, Rockford, IL, USA) supplemented with protease inhibitors (cOmplete, Mini, EDTA-free Protease Inhibitor Cocktail, Roche, Mannheim, Germany) according to the manufacturer instructions. Then, supernatants were aliquoted and stored at −70 °C until used. Western blotting was performed using the Bolt Bis-Tris Plus system (Life Technologies, Carlsbad, CA, USA). Equal amounts of protein (30 μg per well) were separated on gradient gels (Bolt 4–12% Bis-Tris Plus) and electrotransferred onto nitrocellulose membrane (Amersham™ Protran™ 0.45 µm, GE Healthcare, Bensheim, Germany). Then, the membrane was blocked for 15 min in SuperBlock™ (PBS) buffer (Thermo Scientific, Rockford, IL, USA) and incubated overnight with primary antibodies. Next, after washing in PBST and incubating with HRP-conjugated secondary antibodies (Dako, Ely, UK), chemiluminescence was estimated with the ECL Western Blotting Substrate (SuperSignal West Pico Plus, Thermo Scientific, Rockford, IL, USA) on CL-XPosure Film (Agfa Healthcare, Mortsel, Belgium). Then, films were scanned with HP Scanjet G4050 scanner (Hewlett Packard, Palo Alto, CA, USA), and densitometry was determined using ImageJ software version 1.47 (Bethesda, MD, USA). Next, to further analyze other proteins, the membrane was stripped for 10 min in Restore™ PLUS Western Blot Stripping Buffer (Thermo Scientific, Rockford, IL, USA) and re-probed as described above. Primary antibodies were Snail, Slug, vimentin, N-cadherin, E-cadherin and claudin (Cell Signaling Technology, Inc., Beverly, MA, USA), TYMS, and GAPDH (Santa-Cruz Biotechnology, Dallas, TX, USA).

### 2.7. siRNA Transfection

A mixed set of four siRNAs targeting human TYMS and negative control siRNA (scramble) were used (Dharmacon, Lafayette, CO, USA). The siRNAs were introduced into colon cells with Xfect^TM^ Transfection Reagent (Clontech, Mountain View, CA, USA) according to the manufacturer’s protocol, and cells were silenced for 48 h.

### 2.8. Statistical Analysis

All data are expressed as a mean of at least three independent experiments, and the MMP-7 assay was performed in duplicate within an experiment that was repeated three times. The statistical significance of the differences between experiments was determined by Student’s t-test or by ANOVA followed by Tukey’s test. The relationship between the variables was determined by linear regression analysis. All analyses were performed using GraphPad Prism version 9.3 software (GraphPad Inc., San Diego, CA, USA).

## 3. Results

TYMS has been proposed recently to modulate the epithelial–mesenchymal transition in breast and lung cancer. Thus, we checked if TYMS would modulate cell invasiveness properties in colon cancer cells through the stimulation EMT process.

### 3.1. TYMS Protein Level Corresponds with Invasive Potency of Colon Cancer Cells

**Figure 1:**
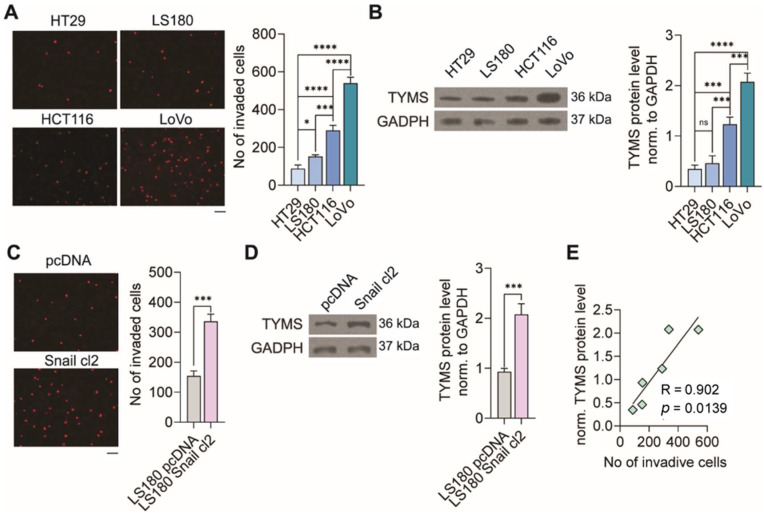
Correlation between TYMS protein level and invasion potential of colon cancer cells. Colon cancer cells (**A**) with different invasiveness properties and (**C**) Snail overexpressing cells were characterized according to their ability to transmigrate through matrigel. The representative images are shown. Scale bar 100 µm. The graphs are displaying means ± S.D. (*n* = 3); * *p* < 0.05, *** *p* < 0.001, **** *p* < 0.0001. Western Blot-measured TYMS protein level in (**B**) CRC panel and (**D**) Snail overexpressing cells. Representative blots are shown, and graphs displaying the mean protein level normalized to GAPDH ± SD (*n* = 3). ns—non-significant; *** *p* < 0.001, **** *p* < 0.0001. (**E**) Linear regression between TYMS protein level and colon cancer cells invasion potential.

Firstly, the panel of CRC cells was characterized according to their invasion ability. Cells were subjected to transwells coated with matrigel, and 8 h later, the number of transmigrated cells was estimated. We noticed that through matrigel transmigrated 1.7, 3.3, and 6.2 times more LS180, HCT116, and LoVo, respectively, than HT29 ones (Figure 1A). Then, we analyzed the TYMS protein level in the studied CRC panel and observed that the TYMS level correlates with the invasive potential of colon cancer cells (Figure 1B).

The highest TYMS protein level was observed in LoVo and the lowest in HT29. Then, we employed the Snail-overexpressing colon cancer cells to confirm that the TYMS protein level corresponds with invasive potency. Snail is a known transcription factor involved in the early EMT process, responsible for developing pro-metastatic phenotype in cancer cells [13].

We applied a previously established colon cancer LS180 cells model stably overexpressing Snail (LS180 Snail cl2) [19]. LS180 pcDNA cells with basal Snail level (stable empty-vector-transfected cells) were used as the control clones. We noticed a 2.2-times-greater number of invaded cells of LS180 Snail cl2 compared to control, empty-vector-transfected cells—LS180 pcDNA (Figure 1C). That result corresponded to a similar growth of TYMS protein level in cells compared to control cells (Figure 1D).

We also performed linear regression and identified a statistically significant (*p* = 0.0139) association between TYMS protein level and invasion potential of studied colon cancer cell lines and Snail-overexpressing cells (Figure 1E).

### 3.2. TYMS Overexpression Induce Epithelial–Mesenchymal Transition in Colon Cancer Cells

**Figure 2:**
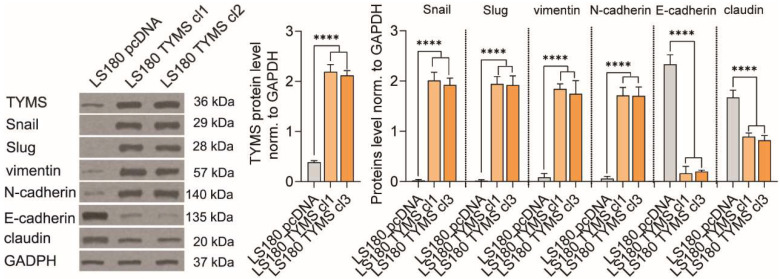
TYMS overexpression induced EMT in LS180 cells. TYMS protein level and EMT markers were measured by Western blot in TYMS overexpressing cells. Representative blots are shown, and graphs displaying the mean protein level normalized to GAPDH ± SD (*n* = 3). ns—non-significant; **** *p* < 0.0001.

Since we observed a correlation between the TYMS protein level and the cells’ ability to invade, we asked whether TYMS itself could have the potency to induce changes in the cell phenotype and increase invasion properties. To verify this, we established preinvasive colon cancer cells stably overexpressing TYMS (LS180 TYMS cl 1 and LS180 TYMS cl 3). We observed that LS180 TYMS cl1 and TYMS cl3 cells had 5.7- and 5.5-fold higher TYMS protein levels than LS180 pcDNA cells, respectively (Figure 2).

Then, we verified whether an increase in TYMS protein level was accompanied by adjustment within the EMT process. Firstly, we analyzed the level of EMT markers and found that TYMS clones revealed higher expression of mesenchymal markers and lower epithelial ones. The level of mesenchymal markers (Snail, Slug, vimentin, and N-cadherin) was proportional to the TYMS protein level, while the level of epithelial markers (E-cadherin and claudin) was inversely proportional to the TYMS (Figure 2).

### 3.3. TYMS Overexpression Induce Invasiveness in Colon Cancer Cells

**Figure 3:**
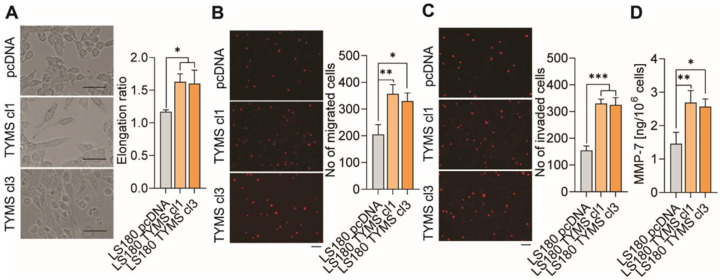
TYMS overexpression cells exhibited an increased invasive phenotype. Cells invasive abilities were evaluated by (**A**) cell elongation, (**B**) cell migration, (**C**) cell invasion assay, and (**D**) MMP-7 secretions. The representative images are shown. Scale bar 100 µm. The graph displays means ± SD (*n* = 3). ns—non-significant * *p* < 0.05, ** *p* < 0.01, *** *p* < 0.001.

Next, as cancer cells undergoing EMT develop an invasive phenotype that is characterized by, among other things, an elongation of the cell shape [20], we studied this feature. We noticed that cell shape was correlated with the TYMS level, and LS180 TYMS cl1 and cl3 cells were 1.39- and 1.37-fold more elongated, respectively, than LS180 control cells (Figure 3A). Furthermore, since cells with increased invasive potential must be characterized by a more remarkable ability to migrate, we investigated the TYMS overexpression on cell transmigration through the membrane to fibronectin chemoattractant. We observed that it migrated 1.74 and 1.61 times more LS180 TYMS cl1 and cl3, respectively, than LS180 pcDNA cells (Figure 3B). Another cardinal feature of cells’ invasive phenotype is their ability to transmigrate through matrigel. We observed that through matrigel transmigrated 2.14 and 2.1 times more LS180 TYMS cl1 and cl3 cells, respectively, than LS180 pcDNA ones (Figure 3C). Additionally, we studied whether TYMS overexpression would affect MMP-7, a small metalloproteinase known to be implicated in colon cancer development by decomposition of the extracellular matrix elements and basement membranes and activation of other MMPs such as proMMP-2 and proMMP-9 [21,22]. To investigate the MMP-7 secretion, we measure protein levels in the medium from TYMS-overexpressing cells. We noticed that MMP-7 protein secretion was correlated with the invasion potential of colon cancer cells. LS180 TYMS cl1 and LS180 TYMS cl3 cells secreted 1.84 and 1.76-fold more MMP-7, respectively, than LS180 control cells (Figure 3D).

### 3.4. TYMS Downregulation Abolishes the EMT Process and Inhibits Colon Cancer Cells Invasion

**Figure 4:**
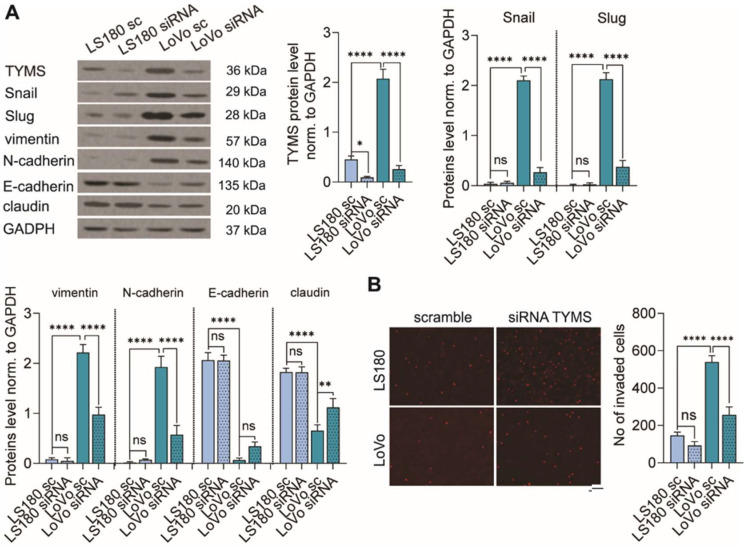
TYMS downregulation inhibited the EMT process and invasion of colon cancer cells. TYMS was downregulated with siRNA in colon cancer cells with different invasiveness properties and (**B**) TYMS overexpressing cells. (**A**) The effectiveness of TYMS-silencing and EMT markers was measured by Western blot. Representative blots are shown, and graphs displaying the mean protein level normalized to GAPDH ± SD (*n* = 3). (**B**) An invasion assay was performed to analyze the effects of TYMS silencing on cell invasive properties. The representative images are shown. Scale bar 100 µm. The graph displays means ± SD (*n* = 3). sc—scramble; siRNA—siRNA targeting human TYMS; ns—non-significant; * *p* < 0.05, ** *p*<0.01, **** *p* < 0.0001.

Hence, we demonstrated that TYMS stimulated the EMT phenotype in preinvasive colon cancer cells and increased the invasive potential of cells, in the next step, we investigated whether TYMS downregulation would inhibit these processes. We successfully diminished TYMS protein level using siRNA targeting human TYMS in LS180 and LoVo cells (reduction of TYMS protein level being around 80% and 88% in LS180 and LoVo, respectively) (Figure 4A). TYMS silencing caused a partial reversion of markers levels. We observed an increasing level of epithelial markers (E-cadherin and claudin) and reduced the mesenchymal ones (Snail, Slug, vimentin and N-cadherin) (Figure 4A). However, it is worth noting that changes were observed only for LoVo cells, which represent a higher invasive phenotype than LS180 cells. We did not observe any effect on LS180 cells. Then, we investigated the role of TYMS downregulation on cell invasiveness. We noticed that TYMS silencing notably inhibited invasion ability in more invasive cells (being 53%) and slightly less, but not statistically significant, in low invasive cells (being 37%) (Figure 4B).

### 3.5. TYMS Silencing Reverses Invasive Phenotype in TYMS-Overexpressing Cancer

**Figure 5:**
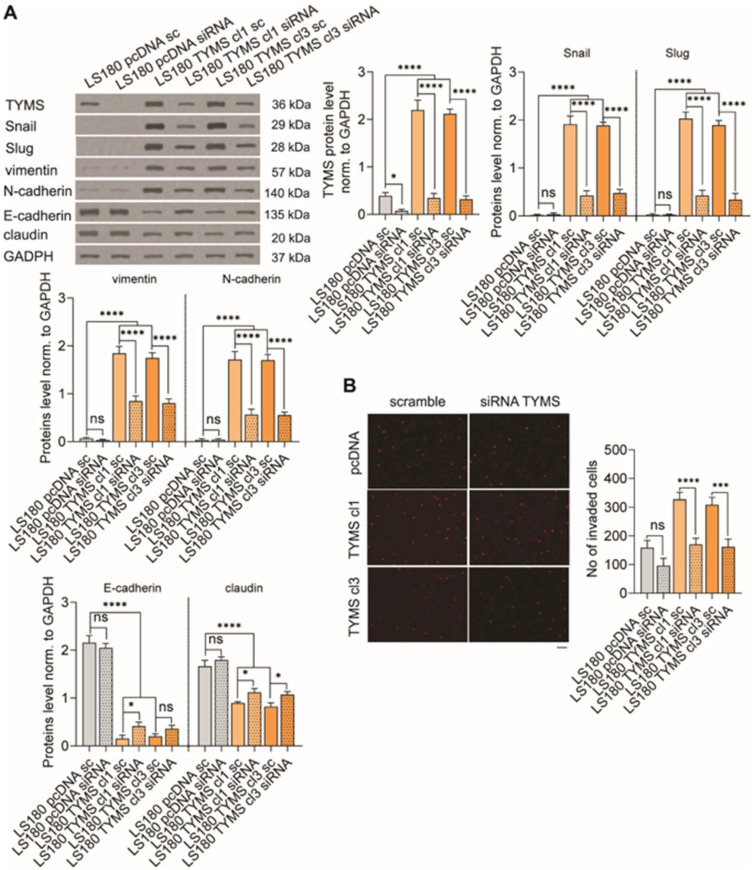
TYMS silencing reversed the EMT process and inhibited the invasion abilities of TYMS-overexpressing cells. TYMS was downregulated with siRNA in TYMS overexpressing cells. (**A**) The effectiveness of TYMS-silencing and EMT markers were measured by Western blot. Representative blots are shown, and graphs displaying the mean protein level normalized to GAPDH ± SD (*n* = 3). (**B**) An invasion assay was performed to analyze the effects of TYMS silencing on cell invasive properties. The representative images are shown. Scale bar 100 µm.The graph displays means ± SD (*n* = 3). sc—scramble; siRNA—siRNA targeting human TYMS; ns—non-significant; * *p* < 0.05, *** *p* < 0.001, **** *p* < 0.0001.

Since TYMS overexpression is often a consequence of using 5-FU chemotherapy in colon cancer treatment [3], we investigated whether TYMS silencing in TYMS-overexpressing cells would reverse the invasive phenotype. Firstly, we notably decreased TYMS protein levels by 81%, 84%, and 85% in LS180 pcDNA, LS180 TYMS cl1, and cl3 cells, respectively, by using siRNA-targeting human TYMS (Figure 5A). Next, we investigated the TYMS silencing effect on EMT, and observed that the process was reversed in TYMS-overexpressing cells. We noticed that mesenchymal markers (like Snail, Slug, vimentin, and N-cadherin) notably decreased, and epithelial markers (E-cadherin and claudin) increased (Figure 5A). However, the changes in epithelial markers were not as spectacular as for mesenchymal ones. It should also be noted that we could not observe any effect of TYMS silencing on EMT markers in LS180 pcDNA cells (Figure 5A). Furthermore, we also investigated whether reversing EMT by TYMS silencing within TYMS-overexpressing cells was accompanied by inhibition of invasion properties. An enormously decreased TYMS protein level inhibited invasion ability by 48% in LS180 TYMS cl1 and LS180 TYMS cl3 cells (Figure 5B). The observed reduction in cells invasion in LS180 pcDNA was 40%. However, it was not statistically significant (Figure 5B).

## 4. Discussion

One of the greatest challenges in treating colorectal cancer is its late diagnosis, which often leads to metastasis and, as a result, patients’ death. It has been estimated that 30% of patients have a too-late diagnosis, and every fourth patient has already metastasized at the time of the first diagnosis of CRC [23]. Therefore, it seems necessary to develop anticancer therapies directed against cancer cells exhibiting a metastatic phenotype.

It is widely accepted that therapies based on 5-FU, currently the primary clinical treatment of colorectal cancer, are very effective, especially in the preinvasive stages. Although using 5-FU in systematic therapy has apparent advantages, patients’ 5-year survival is relatively low [24]. The development of drug resistance due to chemotherapy is estimated as a significant reason. The key factors responsible for the induction of the 5-FU resistance are metabolic enzymes directly or indirectly interacting with 5-FU, and TYMS plays a pivotal role in this process [25,26]. Moreover, it is reported that TYMS is upregulated in response to 5-FU anticancer therapy [3,27,28,29]. Since there is a possible relationship between cancer metastasis and drug resistance, we explored the potential role of TYMS in invasiveness regulation in cancer cells.

Firstly, we analyzed the panel of colon cancer cell lines with different invasion properties and found a correlation between TYMS protein level and cells’ invasion ability. Higher invasive abilities also characterized the cells with higher TYMS protein levels. To confirm that observation, we investigated the TYMS level in cells undergoing epithelial–mesenchymal transition (EMT [14]. The results confirmed our earlier observation—in cells with the Snail overexpression, which induces EMT, we noted an increased level of TYMS protein.

Recent studies revealed the role of TYMS in maintaining EMT in breast and lung cancer cells [10,12]. Therefore, we decided to check in the next step whether the TYMS itself would be able to stimulate EMT in colon cancer cells and, as a consequence, promote invasion ability and then metastasis phenotype. To address this question, we established the TYMS overexpressing colon cancer cells, and we have linked TYMS to EMT and metastasis induction. We noticed that TYMS overexpression induced EMT, manifested by increasing mesenchymal markers and decreasing epithelial ones. Furthermore, TYMS overexpressed cells revealed an elongated shape and a higher ability to migrate, invade and secrete MMP-7 to the extracellular matrix—all the features that make up the metastatic phenotype.

As mentioned before, TYMS upregulation can be stimulated by 5-FU chemotherapy [3,27,28,29] due to the defense cells’ mechanisms. Together with the previous results, our data suggest that 5-FU-induced TYMS could adjust EMT phenotypes, which might develop metastasis phenotypes in patients. Moreover, overall patient survival with higher TYMS levels in primary tumors with metastasis is shorter than in patients with lower TYMS levels after 5-FU-based adjuvant chemotherapy [30]. Hence, considering the above, we asked whether TYMS downregulation might positively impact EMT abrogation and potentially metastasis inhibition. We used two approaches to silencing the TYMS protein. We checked the effect of TYMS downregulation in cells with higher invasive potential and, secondly, in cells with the induced overexpression of TYMS. We observed that the TYMS silencing abolished the EMT process and inhibited the invasion of colon cancer cells. Our results stayed in agreement with the previous ones, which showed that downregulation of TYMS by siRNA resulted in a reversal of the EMT phenotype and inhibition of metastasis in various types of cancer [10,11,12,31]. However, it is essential to note that TYMS downregulation on EMT was observed only in cells characterized by higher invasive potential; the impact on cells with low invasive potential was relatively small and statistically negligible. Since 5-FU chemotherapy might upregulate TYMS, from the clinical point of view, an interesting aspect is whether, in cells with induced expression of TYMS, a reduction of TYMS protein level will lead to a reversal of the EMT. Indeed, we observed that TYMS diminished could reverse the invasive phenotype in TYMS-overexpressing colon cancer cells. This observation might have a beneficial consequence in establishing new anticancer therapy against metastatic tumors. Despite the promising research in the cellular model, we are aware that the analysis should be extended to animal studies, particularly regarding the 5-FU resistance model with induced expression of TYMS. A further in-depth investigation is needed to verify whether TYMS inhibitor would abrogate the invasion properties of colon cancer cells and be able to reverse the EMT phenotype stimulated by 5-FU treatment.

In summary, we established the role of TYMS in metastasis modulation of colon cancer cells through EMT stimulation. We showed both that TYMS overexpression drives the invasive phenotype and TYMS silencing abrogated the invasion abilities. This observation strongly indicates that TYMS could be an attractive target against the metastatic sites of colon cancer occurring as a result of 5-FU chemotherapy.

## Data Availability

The data presented in this study are available on reasonable request from the corresponding author.
